# A Case Report and Literature Review on Osseous Metaplasia in Traditional Serrated Adenoma

**DOI:** 10.7759/cureus.39770

**Published:** 2023-05-31

**Authors:** Jamila Mammadova, Katsiaryna Khatskevich, Chadi Hajar

**Affiliations:** 1 Department of Hematology and Oncology, University of South Florida, Tampa, USA; 2 Department of Pathology and Laboratory Medicine, Medical University of South Carolina, Charleston, USA

**Keywords:** traditional serrated adenoma, osseous metaplasia, gastrointestinal tract, colorectal polyp, mesenchymal metaplasia

## Abstract

Osseous metaplasia is an extremely rare occurrence in traditional serrated adenoma (TSA). We report a case of a 50-year-old female with a TSA with osseous metaplasia (OM). The adenoma was identified during a colonoscopy for endoscopic mucosal resection of a previously identified polyp. The polyp location was the rectum. The colonoscopy was negative for any signs of concurrent malignancy. This case report is the fifth case of OM in a TSA reported in English. The clinical significance of OM is uncertain, and there is limited literature describing these lesions.

## Introduction

Traditional serrated adenomas (TSA) account for less than 1% of colorectal polyps and 1%-7% of all serrated lesions [1]. Osseous metaplasia (OM) in these polyps is rare. Metaplastic lesions represent a replacement of one type of adult cells with another. This process may stem from a biological adaptation to stress through the recruitment of resilient cell types. OM is a process of transformation of cells of fibrous connective tissue into bone-forming cells, which results in the deposition of mature, lamellar bone in abnormal locations [2]. 

OM was first described in gastric adenocarcinoma by Gruber in 1913, followed by the description of OM in four cases of colonic adenocarcinomas by Dukes in 1939 [3,4]. While OM in benign, pre-malignant, and malignant lesions have been previously described, OM in TSAs remains an extremely rare finding. We report a case of a 50-year-old female with a TSA with OM. Fewer than five cases of TSA with OM were reported in the English literature.

## Case presentation

A 50-year-old Caucasian woman was referred for colonoscopy for endoscopic mucosal resection of a previously identified polyp. Her past medical history includes gastroesophageal reflux disorder (GERD), hypertension, and hypothyroidism. One polyp with a diameter of 3.5 cm was resected from the rectum.

**Figure 1:**
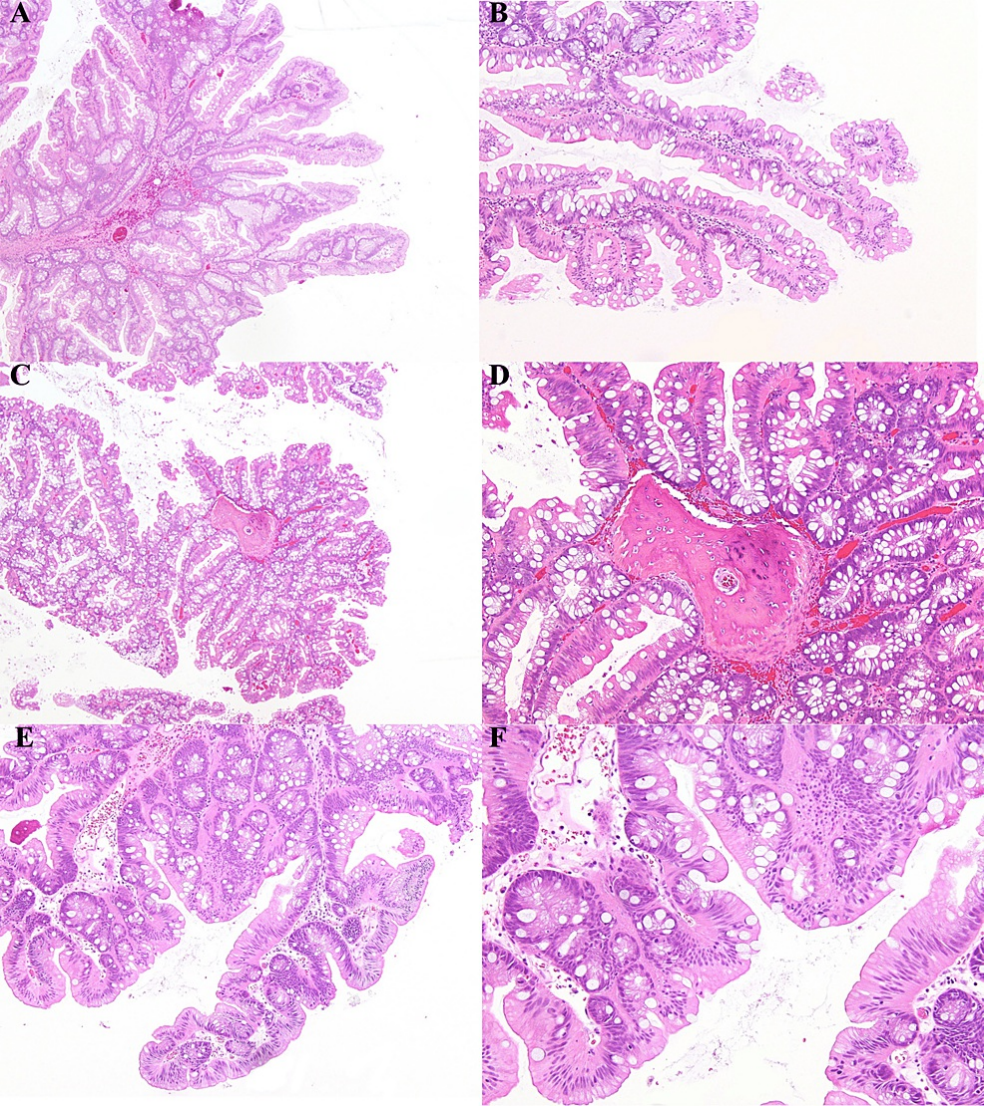
Traditional serrated adenoma with osseous metaplasia All histologic samples were stained with hematoxylin and eosin. A and B: Traditional serrated adenoma (TSA) of rectal mucosa with filiform architecture and ectopic crypts, 40× and 100×, respectively. C and D: Foci of osseous metaplasia in a TSA, 40× and 100×, respectively. E and F: Section demonstrating low-grade dysplasia within columnar cells showing eosinophilic cytoplasm, 100× and 200×, respectively.

A biopsy of the tissue revealed a large high rectal adenoma, particularly a TSA with OM. The polyp demonstrated a villiform growth pattern with pseudostratified eosinophilic columnar cells and dark, penicillate dysplastic nuclei. Slit-like seriations and foci of ectopic crypts were noted. There was no observed loss of polarity, nuclear enlargement, pleomorphism, glandular crowding, or malignant glands with desmoplastic reaction excluding high-grade dysplasia or invasive carcinoma. Colonoscopy revealed an otherwise healthy colon without additional polyps. No other metaplastic changes were noted by staining with hematoxylin and eosin (H&E) including squamous or clear cell metaplasia. The images of the histology slides can be seen in Figure 1.

## Discussion

OM is a type of mesenchymal metaplasia that constitutes a rare phenomenon for gastrointestinal tract (GIT) neoplasms. It is defined by the presence of benign bone tissue that forms in a location where it would not normally be found. It is described more commonly for colorectal carcinomas and their metastases. OM in benign lesions, particularly in TSAs, is an extremely rare finding with less than five reported cases (Table 1). 

**Table 1:** Summary of the current case and previously reported cases of osseous metaplasia in traditional serrated adenoma FOBT: fecal occult bleed test; ND: no data; TSA: traditional serrated adenoma

First author (Ref)	Reported year	Age, years	Sex	TSA location	Size, cm	Identification	Grade of dysplasia
Mammadova (current issue)	2023	50	F	Rectum	3.5	Colonoscopy for endoscopic mucosal resection of polyp	Low-grade
Smith [5]	2021	29	F	Rectum	ND	Colonoscopy for investigation of a 3-year history of fresh rectal bleeding and diarrhea	Low-grade and small foci of high-grade
Montalvo [6]	2012	62	M	Sigmoid colon	5 × 3.5 × 2	Routine endoscopic examination	Low-grade
Wilsher [7]	2011	66	F	Left colon	1.3 x 1.3 x 1.1 and 2.5 x 1.0 x 1.2	Colonoscopy following a positive FOBT	Both low- and high-grade
Wilsher [8]	2010	50	M	Colon	2.5	Colonoscopy following a positive FOBT	Patchy, low-grade

The clinical significance of OM in GIT neoplasms remains uncertain. Previously, OM was described to be associated with symptomatic bleeding and was considered a high-risk feature [9]. In our literature review, three out of four reported cases of TSAs with OM had either overt or occult rectal bleeding. Our patient did not present with symptoms of bleeding. The rare occurrence and scarce literature description of these lesions constitute limitations in interpreting their clinical relevance. Additionally, OM within adenocarcinomas may be falsely interpreted as sacral invasion, which also raises some clinical relevance [8]. The continuous reporting of additional factors, such as dysplasia grade, size, staining, symptoms at presentation, and malignant transformation may improve understanding of the clinical significance of TSAs with OM.

For the previously reported four cases, the sites of TSA were the left colon (3/4) and rectum (1/4). In this case, the location of TSA was the rectum. The malignant transformation with the development of adenocarcinoma occurred in one of the four reported cases [7]. For our patient, no sign of malignancy was noted.

The exact mechanism underlying OM is not fully understood, but several hypotheses exist in the literature. The ability of fibroblasts to undergo a transformation into osteoblasts may constitute the basis of such metaplasia [10]. The subsequent synthesis of bone tissue by osteoblast-like cells was previously postulated based on increased expression of alkaline phosphatase [11]. Increased expression of bone morphogenetic proteins (BMP) expressed by epithelial cells and fibroblasts was previously detected in OM of colorectal adenocarcinomas. Kypson et al. compared the expression of BMP-2 in rectal adenocarcinoma with and without OM. The expression was higher in rectal adenocarcinomas with OM, and the authors suggested that the stimulus may come from epithelial cells [12]. In another study, BMP-2, BMP-4, BMP-5, and BMP-6 were detected in osteoblast-like cells and tumor cells, and surrounding stromal fibroblasts expressed BMP-2 and BMP-4 [13]. The exact mechanism remains uncertain.

## Conclusions

This case report describes a rare finding of osseous metaplasia (OM) in traditional serrated adenoma. To the best of the authors' knowledge and based on an extensive literature review, this is the fifth case of OM in this specific type of colorectal polyp. Although it is a benign finding, additional data analysis is needed to understand the exact pathophysiologic mechanism and clinical significance.

## References

[1] McCarthy AJ, Serra S, Chetty R (2019). Traditional serrated adenoma: an overview of pathology and emphasis on molecular pathogenesis. BMJ Open Gastroenterol.

[2] Fernandes TR, Grandi F, Monteiro LN, Salgado BS, Rocha RM, Rocha NS (2012). Ectopic ossification presenting as osteoid metaplasia in a salivary mucocele in a Shih Tzu dog. BMC Vet Res.

[3] Gruber GB (1913). Knochebildung in einem magen karzinom (Article in German). Z Beitr Path Anat.

[4] Dukes CE (1939). Ossification in rectal cancer. Proc R Soc Med.

[5] Smith G, Laskaratos FM, Mitchison M, Vega R (2021). A rare endoscopic finding of a traditional serrated adenoma with osseous metaplasia in the rectum. Clin Res Hepatol Gastroenterol.

[6] Montalvo NF, Beltrán JN, Redrobán LA (2012). Traditional serrated adenoma of the sigmoid colon with osseous metaplasia: a case report. J Med Case Rep.

[7] Wilsher MJ (2011). Adenocarcinoma arising in a traditional serrated adenoma of the rectosigmoid colon with osseous metaplasia. Pathol Int.

[8] Wilsher MJ, Mendelsohn GB (2010). Osseous metaplasia in a traditional serrated adenoma of the rectosigmoid colon. Pathology.

[9] Bowman EA, Stevens EC, Pfau PR, Spier BJ (2012). Ossification within an adenomatous polyp: a case report and review of the literature. Eur J Gastroenterol Hepatol.

[10] Marks MM, Atkinson KG (1964). Heterotopic bone in a juvenile rectal polyp: case report. Dis Colon Rectum.

[11] Randall JC, Morris DC, Tomita T, Anderson HC (1989). Heterotopic ossification: a case report and immunohistochemical observations. Hum Pathol.

[12] Kypson AP, Morphew E, Jones R, Gottfried MR, Seigler HF (2003). Heterotopic ossification in rectal cancer: rare finding with a novel proposed mechanism. J Surg Oncol.

[13] Imai N, Iwai A, Hatakeyama S (2001). Expression of bone morphogenetic proteins in colon carcinoma with heterotopic ossification. Pathol Int.

